# MIMOX: a web tool for phage display based epitope mapping

**DOI:** 10.1186/1471-2105-7-451

**Published:** 2006-10-12

**Authors:** Jian Huang, Alex Gutteridge, Wataru Honda, Minoru Kanehisa

**Affiliations:** 1 Bioinformatics Center, Institute for Chemical Research, Kyoto University, Uji, Kyoto 611-0011, Japan; 2 School of Life Science and Technology, University of Electronic Science and Technology of China, China

## Abstract

**Background:**

Phage display is widely used in basic research such as the exploration of protein-protein interaction sites and networks, and applied research such as the development of new drugs, vaccines, and diagnostics. It has also become a promising method for epitope mapping. Research on new algorithms that assist and automate phage display based epitope mapping has attracted many groups. Most of the existing tools have not been implemented as an online service until now however, making it less convenient for the community to access, utilize, and evaluate them.

**Results:**

We present MIMOX, a free web tool that helps to map the native epitope of an antibody based on one or more user supplied mimotopes and the antigen structure. MIMOX was coded in Perl using modules from the Bioperl project. It has two sections. In the first section, MIMOX provides a simple interface for ClustalW to align a set of mimotopes. It also provides a simple statistical method to derive the consensus sequence and embeds JalView as a Java applet to view and manage the alignment. In the second section, MIMOX can map a single mimotope or a consensus sequence of a set of mimotopes, on to the corresponding antigen structure and search for all of the clusters of residues that could represent the native epitope. NACCESS is used to evaluate the surface accessibility of the candidate clusters; and Jmol is embedded to view them interactively in their 3D context. Initial case studies show that MIMOX can reproduce mappings from existing tools such as FINDMAP and 3DEX, as well as providing novel, rational results.

**Conclusion:**

A web-based tool called MIMOX has been developed for phage display based epitope mapping. As a publicly available online service in this area, it is convenient for the community to access, utilize, and evaluate, complementing other existing programs. MIMOX is freely available at .

## Background

Since the pioneering work of Smith and co-workers [1-3], phage display technology has been widely used in both basic research such as the exploration of protein-protein interaction sites and networks [2-5], and applied research such as the development of new drugs, diagnostics, and vaccines [6-8]. Phage display has also become a promising epitope mapping method, which has been applied in many fields such as allergology[9] and oncology[10]. The phage display based epitope mapping is usually accomplished through comparing the sequence of mimotopes (antibody-selected phage displayed peptides) to the antigen. In some cases, the mimotope sequence is identical or very similar to a sequence in the antigen[2], there by indicating the location of the native epitope. These cases are rare however, and usually the mimotope sequence has little, if any, similarity with the antigen sequence. Compared with traditional epitope mapping methods such as solving the crystal structure of the antigen-antibody complex or scanning overlapping peptides of the antigen, phage display based epitope mapping is generally much cheaper and less arduous.

Though epitope mapping based on phage display can be done manually[11], it is quite tedious and time-consuming to compare a set of mimotopes to the antigen without computational support. The low sequence similarity between the mimotope and the antigen often makes the mapping even harder. To solve these problems, several groups have researched algorithms and programs that assist and automate phage display based epitope mapping [12-17]. According to their dependency on antigen structure, the existing programs for phage display based epitope mapping can be classified into three categories. Program in the first category such as FINDMAP, only work with sequence data from the mimotopes and antigen[13]. The second category needs both the sequence data and the antigen structure. SiteLight[12], 3DEX[14], and Mapitope[16,17] belong to this category. A very recently published work: MIMOP[15] makes the third category, which integrates the two different approaches and can work with or without the antigen structure. Though implemented differently, all the existing programs have succeeded in given cases. However, most of the existing tools have not been implemented as a freely available online service until now, making it less convenient for the community to access, utilize, and evaluate them.

In the present study, we describe a web-based tool for phage display based epitope mapping named MIMOX. It was coded with Perl as a CGI program and can be used to align a set of mimotopes and derive a consensus sequence. The consensus sequence, or a single mimotope sequence, can then be mapped on to the antigen structure, and potential epitopes determined by spatial clustering of the mapped residues. The results mapped on to the antigen's 3D structure can then be viewed interactively. To validate this web-based tool, we compared the results from MIMOX with the results from other computational tools and experimentally identified native epitopes in several case studies.

## Implementation

### Overall architecture of MIMOX

**Figure 1:**
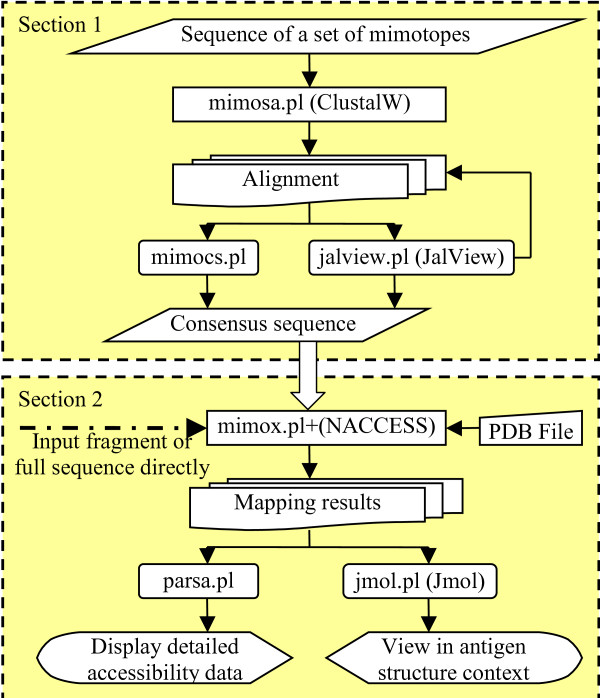
Overall architecture of MIMOX. MIMOX has two sections. The first section has 3 perl scripts. The script *mimosa.pl *aligns a set of mimotope sequences powered by ClustalW. The script *jalviews.pl *wraps JalView to view and manage the alignment. The script *mimocs.pl *derives a consensus sequence from the alignment. The second section also has 3 perl scripts. The script *mimox.pl *maps the user supplied sequence on to the given antigen structure and utilizes NACCESS to calculate the accessibility. The script *parsa.pl *displays the detailed accessibility information of each mapping result. The script *jmol.pl *wraps Jmol to view the mapping result interactively on the antigen structure.

MIMOX was coded with Perl using modules from the Bioperl[18] project. The whole online service provided by MIMOX is accomplished through a set of CGI scripts. The MIMOX service can be divided into two main sections. In the first section, MIMOX provides a simple interface for ClustalW[19] to align a set of mimotope sequences; this is implemented as the script *mimosa.pl*. The alignment can then be used to derive the consensus sequence through a simple statistical method; this is implemented as the script *mimocs.pl*. The alignment can also be viewed and managed through an embedded Java applet version of JalView[20]; this is implemented as the script *jalviews.pl*. In the second section, MIMOX tries to map the user supplied sequence on to the given antigen structure. This is implemented as the script *mimox.pl*. The program NACCESS[21] is also wrapped into *mimox.pl *and used to calculate the surface accessibility of the mapping results. All mapping results are ranked based on their solvent accessible surface. Each mapping result has detailed information of the accessibility of each candidate residue, which is parsed through the script *parsa.pl *and displayed as a table in a new window. Each mapping result can also be viewed interactively on the antigen structure. This is implemented as the script *jmol.pl*, which wraps a Java applet version of Jmol[22]. The overall architecture of MIMOX is shown schematically in Figure 1.

### Deriving consensus sequence from a set of mimotopes

**Figure 2:**
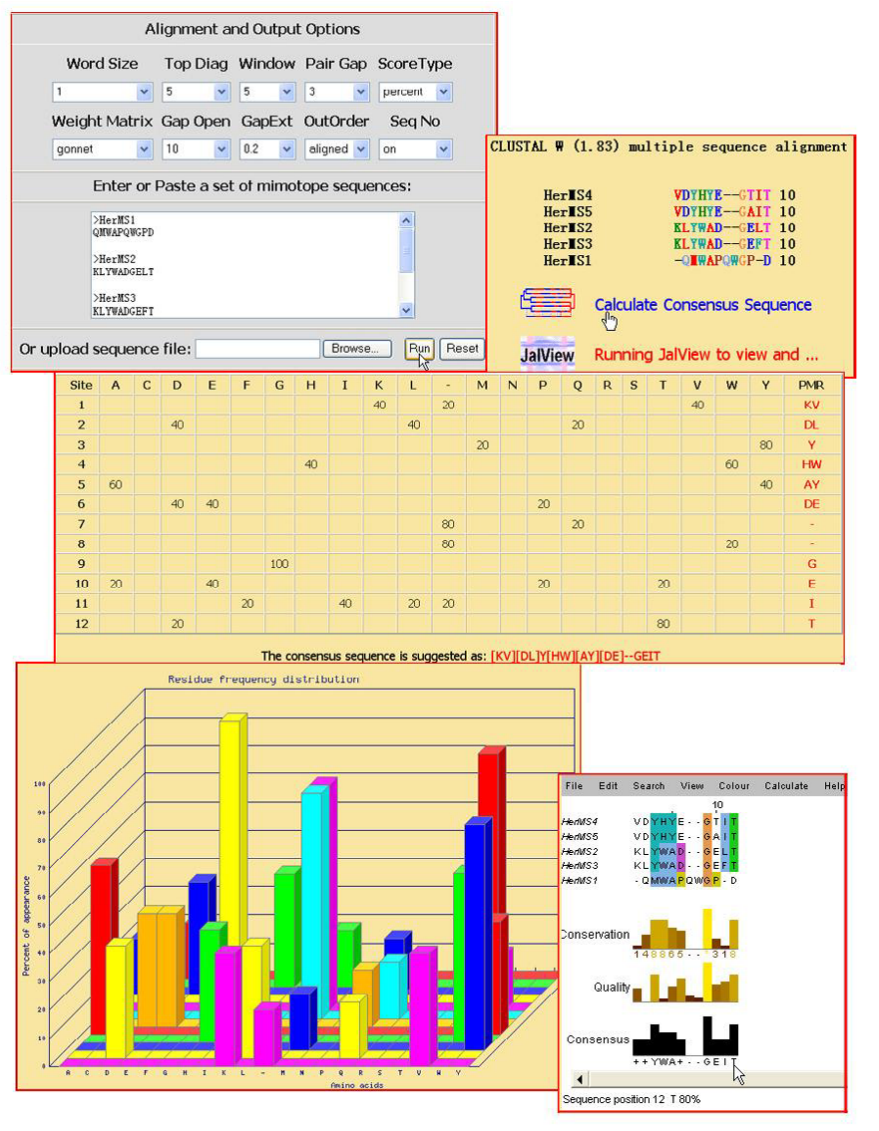
Web interface of MIMOX section 1. Mimotopes selected out with trastuzumab[10] are input and aligned with wrapped ClustalW. The frequency of a given amino acid at each position of the alignment is calculated and displayed in a table. A 3D bar figure is also created, where the X axis represents the 20 amino acid types and gap, Y axis is the frequency and Z axis stands for the position of the aligned sequences. A consensus sequence is then suggested, which can be used in further mapping with MIMOX. The alignment can also be managed with the embedded JalView[20].

As described above, MIMOX wraps ClustalW to align a set of mimotope sequences and then allows the alignment to be viewed, edited, and analyzed through an embedded version of JalView. Based on the review by Smith et al [3], we also implemented a simple statistical method in the script *mimocs.pl *to derive a consensus sequence from the alignment. Firstly, the script counts the appearance of each kind of amino acid at each position in the alignment and calculates the percentage frequency of each one. The frequency of a given amino acid X at the position *i *of the alignment (*f*_*xi*_) is defined as

fxi=XiN×100%
 MathType@MTEF@5@5@+=feaafiart1ev1aaatCvAUfKttLearuWrP9MDH5MBPbIqV92AaeXatLxBI9gBaebbnrfifHhDYfgasaacH8akY=wiFfYdH8Gipec8Eeeu0xXdbba9frFj0=OqFfea0dXdd9vqai=hGuQ8kuc9pgc9s8qqaq=dirpe0xb9q8qiLsFr0=vr0=vr0dc8meaabaqaciaacaGaaeqabaqabeGadaaakeaacqWGMbGzdaWgaaWcbaGaemiEaGNaemyAaKgabeaakiabg2da9maalaaabaGaemiwaGLaemyAaKgabaGaemOta4eaaiabgEna0kabigdaXiabicdaWiabicdaWiabcwcaLaaa@3B8F@

where *Xi *means the times that the given amino acid X appears at the position *i *of the alignment and N is the number of sequences in the alignment. All frequencies are compared to a threshold value, which is 25% by default. If a frequency is more than the threshold, the corresponding residue is considered as a motif residue at that position. If the sum frequency of similar residue at the same position is above the threshold, the similar residues are also regarded as motif residues. In MIMOX, there are five similar residue groups (L, I, V; T, S; E, D; Q, N; K, R; F, W); other residues are considered unique. This classification scheme is the same as that used by Mapitope[16,17]. If no motif residue is found at a given position, then X is used to stand for any amino acid residue. Motif residues at all positions of the alignment are then displayed in a table. Thus a consensus sequence is suggested by the program. The script also creates a 3D bar figure based on the statistical analysis above, where the X axis represents the 20 amino acid types and gap type, Y axis is the frequency and Z axis stands for the position of the aligned sequences (Shown in Figure 2).

### Mapping sequence to the corresponding antigen structure

Since the mimotopes and the native epitope on the antigen bind to the same antibody, it is assumed that the mimotopes and the native epitope have similar physicochemical properties and similar spatial organization. This assumption is the basis of the MIMOX algorithm. The mapping process of MIMOX is based on the input sequence (such as the consensus sequence) and the uploaded antigen structure. A fragment of the sequence can also be used as input.

Firstly, for each position in the input sequence, MIMOX searches the uploaded PDB structure for matching residues and places them into an array of candidate residues for that position. Two matching modes are available at present. One is strict mode, which means the type of mimotope residue must match the antigen residue exactly. The other is called conservative mode, which means similar residues are also included in the candidate residue array. There are 5 groups of similar residues (L, I, V; T, S; E, D; Q, N; K, R; F, W) in MIMOX, which has been described in previous section.

The array of candidate residues for each position is then added to an array of arrays. MIMOX finds all the residue neighbour pairs between consecutive candidate residue arrays in the array of arrays. Whether two residues are neighbours is determined by the distance between the two residues and the distance threshold value. If the distance between two residues is below the threshold, the two residues are taken as a neighbour pair. MIMOX provides three methods to calculate neighbour residue pairs. One method is to take the distance between the Cα atoms of the two amino acids as the distance between the two residues. Using Cα atoms may better reflect the backbone positions. The second method is to use the distance between the Cβ atoms, which may better reflect the side chain position (Cα atom is still used when it is a glycine because it does not have a Cβ atom). The third method described below, is based on the distances between all the heavy atoms of the two amino acids. All of the distances mentioned above are Euclidean distances, calculated as:

D21=(x2−x1)2+(y2−y1)2+(z2−z1)2
 MathType@MTEF@5@5@+=feaafiart1ev1aaatCvAUfKttLearuWrP9MDH5MBPbIqV92AaeXatLxBI9gBaebbnrfifHhDYfgasaacH8akY=wiFfYdH8Gipec8Eeeu0xXdbba9frFj0=OqFfea0dXdd9vqai=hGuQ8kuc9pgc9s8qqaq=dirpe0xb9q8qiLsFr0=vr0=vr0dc8meaabaqaciaacaGaaeqabaqabeGadaaakeaacqWGebardaWgaaWcbaGaeGOmaiJaeGymaedabeaakiabg2da9maakaaabaGaeiikaGIaemiEaG3aaSbaaSqaaiabikdaYaqabaGccqGHsislcqWG4baEdaWgaaWcbaGaeGymaedabeaakiabcMcaPmaaCaaaleqabaGaeGOmaidaaOGaey4kaSIaeiikaGIaemyEaK3aaSbaaSqaaiabikdaYaqabaGccqGHsislcqWG5bqEdaWgaaWcbaGaeGymaedabeaakiabcMcaPmaaCaaaleqabaGaeGOmaidaaOGaey4kaSIaeiikaGIaemOEaO3aaSbaaSqaaiabikdaYaqabaGccqGHsislcqWG6bGEdaWgaaWcbaGaeGymaedabeaakiabcMcaPmaaCaaaleqabaGaeGOmaidaaaqabaaaaa@4DC9@

where *D*_21 _means the distance between atom 2 and atom 1 and *x*_2_*, y*_2_*, z*_2_*, x*_1_*, y*_1_*, z*_1 _are coordinates of atom 2 and atom 1. When the methods based on Cα or Cβ atom position are used, the default distance threshold is 7.0 angstroms, as it approximates the upper limit for noncovalent interactions in macromolecular structures[23]. When the third method is used, the distance threshold is calculated as

*DT *= *DF *× *(vdwAtom1 + vdwAtom2)*

where *DT *is the distance threshold, *DF *is the Distance Factor (given by user), and *vdwAtom *is the *Van der Waals *radius of the atom. The default DF value is 1.11. If two residues have a pair of heavy atoms which are nearer than the distance threshold calculated from their *Van der Waals *radius, the two residues are taken as a neighbour pair.

MIMOX then recursively links the neighbour pairs until all possible ways of forming the input sequence are made. Each result is then ranked according to the sum of the absolute residue accessibility of each residue calculated from the NACCESS result file. In the end, the results are displayed in a table with hyperlinks to call the script *parsa.pl *which can parse and display the accessibility data in detail, and the script *jmol.pl *to view the result interactively mapped onto the antigen structure.

## Results and discussion

### Web interface of MIMOX

**Figure 3:**
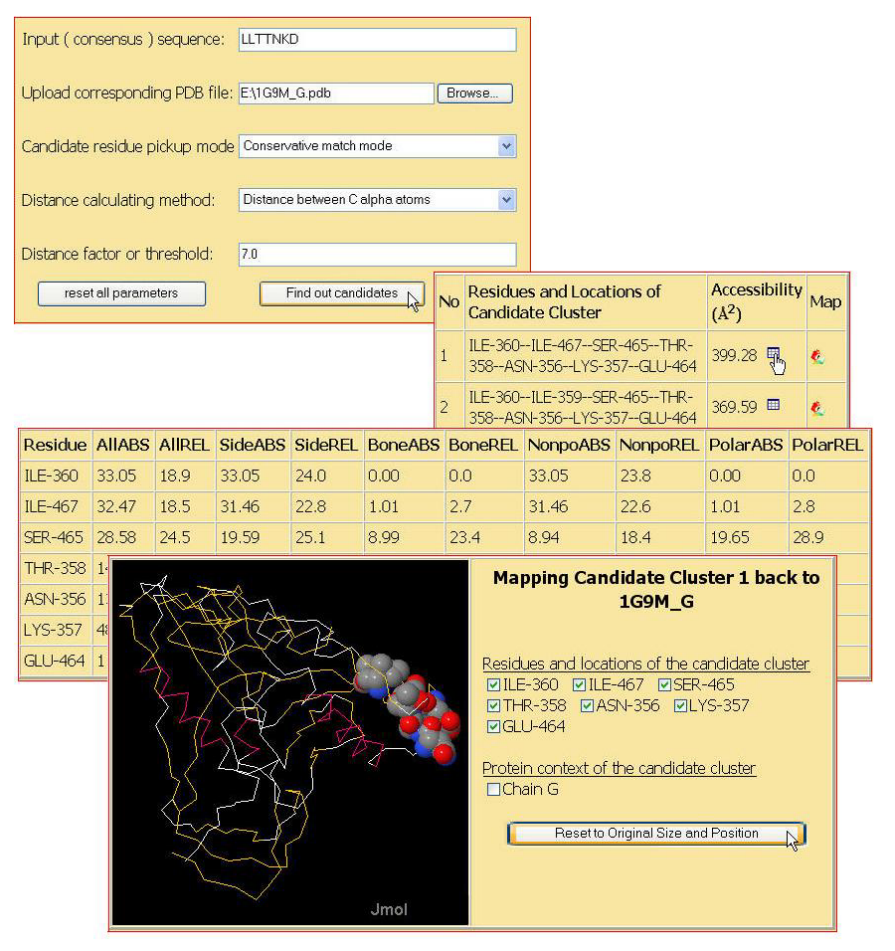
Web interface of MIMOX section 2. LLTTNKD[14] is mapped to the HIV gp120 envelope glycoprotein via MIMOX. When picking candidate residues in conservative mode and all other parameters in defaults, a new top result is suggested. The detailed accessibility data and the image of the new mapping result are dispaleyed accordingly.

MIMOX has successfully been implemented as an online service, which has a simple web interface both for input and output. As described previously, MIMOX can be divided into two sections; we show here the input and output of the two sections in Figure 2 and Figure 3 respectively.

### Case studies

To test MIMOX, we have applied it to several cases taken from other similar research and literature. We compared the results from MIMOX with the results from other computational tools and the native epitope itself if the epitope is known in the CED database[24]. It should be pointed out that cases using monoclonal antibodies are most appropriate for testing[15]. However, in order to compare with previously published tools, some less appropriate cases (using polyclonal antibodies) taken from the corresponding literature are also used. More case studies [see Additional file 1] can also be found on the test dataset page of MIMOX[25].

The first case is taken from FINDMAP[13]. In 1999, Jesaitis and co-workers used an anti-actin polyclonal antibody to select a phage displayed random peptide library; VPHPTWMR was one of the consensus sequences they derived from the selected mimotopes. They manually mapped VPHPTWMR to the known structure of actin [PDB: 1ATN] and suggested that it might correspond to residues: V129, P130, H101, P102, T358, W356, M355, R372[11]. In 2003, Mumey et al used FINDMAP to align VPHPTWMR to the actin sequence without utilizing information on the antigen structure. The result from FINDMAP shows VPHPTWMR can be mapped to residues as V129, P130, H101, P102, T103, W356, M355, R372[13]. FINDMAP mapped the input sequence to a slightly different set of residues (using T103 instead of T358). When running MIMOX with all parameters as defaults, we got no result. However, after the distance threshold is changed to 12 Å (the maximum distance allowed in MIMOX), we find that the two mappings above are returned as candidate cluster 5 and candidate cluster 17. As the side chain of some amino acids (such as arginine) can span a distance as great as 12 Å, MIMOX takes this value as the maximum allowable distance. This distance restriction is also used by Mapitope[16,17]. In this case, the need for the higher distance threshold is due to R372 which lies some distance from the other mapped residues. MIMOX also suggested other possibilities such as cluster 1(V96, P102, H101, P130, T358, W356, M355, R372) which has a bigger solvent accessible surface, and cluster 26 (V96, P98, H101, P102, T103, W356, M355, R372), which clearly has 3 sequential segments, i.e. VPHPT, WM, and R.

The second case is taken from work by Enshell-Seijffers[16]. They used monoclonal antibody 17b, which is against HIV gp120 envelope glycoprotein, to select a phage displayed random peptide library and got a set of 11 mimotopes. Analyzing the mimotopes with Mapitope, they suggest that the 17b epitope might consist of the following residues and segments: L111, LKPCVK (116–121), P124, VITQ (200–203), CPKV (205–208), RIK (419–421), I423, I424, K432, P437, P438. The structure of HIV gp120 envelope glycoprotein in complex with 17b has been solved [PDB: 1GC1] and 17b epitope has been recorded in the CED database as CE0058, which is composed of CK(119,121) + VTQAC(200,202–205) + RKQI(419,421–423) + KMYP (432,434,435,437). Among the 24 Mapitope predicted residues, 11 are contact residues of the 17b epitope. Using default parameters, MIMOX derived a consensus sequence, [LV] RP [LT] [KR] LRE [LP] [RT] X [-R], from 17b mimotopes. MIMOX finds no result matching the whole consensus sequence. Using LRLR, a fragment of the consensus sequence as the input sequence and running MIMOX in conservative match mode with other parameters as defaults, the top result is I423, K421, I420, and R419; the 4th result is V200, K121, L122, K432 and the 5th result is I423, K432, L122, and K121. Taking the top 5 results together, 13 residues are suggested by MIMOX and 6 of them are contact residues of the 17b epitope. In this case, Mapitope gives more complete and elaborate result.

**Figure 4:**
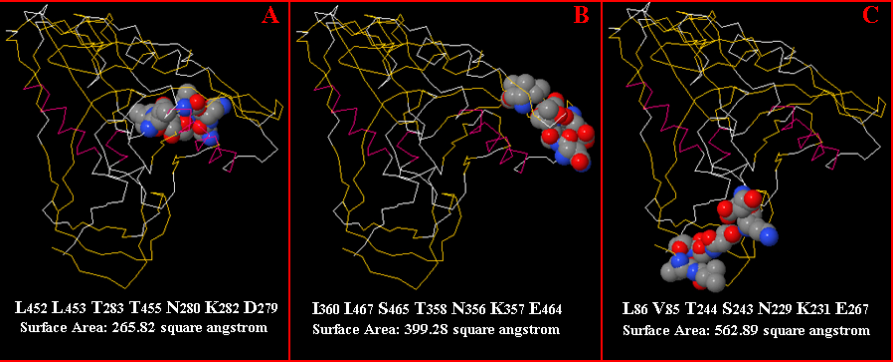
Comparison of three mapping results. MIMOX was used to map LLTTNKD to HIV gp120 with three different methods. The top result using the default parameters is shown in image A. It is composed of the residues: L452 L453 T283 T455 N280 K282 D279 and has a 265.82 Å^2 ^solvent accessible surface. The top result from conservative mode is shown in image B. It is composed of the residues: I360 I467 S465 T358 N356 K357 E464, with a 399.28 Å^2 ^surface area. Image C shows the top result from the all heavy atoms method. It is composed of the residues: L86 V85 T244 S243 N229 K231 E267 and has an even larger accessible surface area of 562.89 Å^2^.

The third case is taken from 3DEX[14]. LLTTNKD is a mimotope selected from a phage displayed random peptide library using HIV positive patients' IgG. Using 3DEX to map this mimotope to the HIV gp120 envelope glycoprotein [PDB: 1G9M], Schreiber et al reported that this mimotope might correspond to residues: L452, L453, T283, T455, N280, K282, and D279. When running MIMOX with default parameters, the top candidate residue cluster is exactly the same as the result of 3DEX, which has a 265.82 Å^2 ^solvent accessible surface. By picking candidate residues in conservative mode and clustering based on Cα atoms with a distance threshold 7 Å, the top candidate residue cluster suggested by MIMOX is changed to I360, I467, S465, T358, N356, K357, and E464. This cluster has a 399.28 Å^2 ^accessible surface area. Furthermore, when neighbouring based on all heavy atoms with a distance factor 1.11, the top candidate residue cluster suggested by MIMOX is L86, V85, T244, S243, N229, K231, and E267, which has an even larger accessible surface area, 562.89 Å^2^. All 3 clusters are shown in Figure 4. As the latter two mapping results suggested by MIMOX are more exposed, they might be able to bind to the antibody more easily.

The last case is taken from MIMOP[15]. BO2C11 is a human monoclonal antibody against human coagulation factor VIII. Villard et al selected two phage displayed random peptide libraries with BO2C11 and got a set of 27 mimotopes[26]. Very recently, Moreau et al have applied their newly developed tool MIMOP to analyze these mimotopes. Combining the two methods MimAlign and MimCons in MIMOP, the BO2C11 epitope is predicted be composed of a segment YFTNMF (2195–2200) and residues T2202, K2207, R2215, R2220, Q2222. The structure of human coagulation factor VIII in complex with BO2C11 has been solved [PDB: 1IQD] and the BO2C11 epitope has been recorded in the CED database as CE0176, which consists of FTNMF (2196–2200), R2215, RPQV (2220–2223), SLLT (2250–2253), HQ (2315–2316). Using default parameters, MIMOX derived a consensus sequence of [NQKR] [HST] RWSNRSS [ST] from those mimotopes. Again, the whole length consensus sequence returns no mapping results. However, when we use QH, RWSN, RSSS, three sequential fragments that cover the whole consensus sequence, as input sequences and running MIMOX in conservative match mode with all other parameters as defaults, the top 3 results of each of the partial sequences overlap with the MIMOP result and the native BO2C11 epitope well. For example, the third result of the input QH suggested by MIMOX is Q2316, H2315; the third result of the input RWSN suggested by MIMOX is R2220, F2196, T2197, N2198; the first result of the input RSSS suggested by MIMOX is R2215, S2216, T2202, T2197 and third result is K2249, S2250, S2254, T2253.

Taking together, our initial case studies show that MIMOX can fully or partially repeat results from manual mapping, other existing tools, and also provide novel suggestions. MIMOX is designed to be a tool which is more interactive than automatic. We acknowledge that tuning the probe sequences and parameters are often required to get good results. This interactive process gives hints to users step by step and greatly decreases the load of the server and prevents the loss of some reasonable results. MIMOX lists all the matched results with no prediction threshold. This allows users to find the reasonable results by themselves based on their background knowledge on a given antibody, a given antigen and a given phage display experiment. Nevertheless, according to the test dataset page of MIMOX, the true epitope (or its segments) often falls in the top 5 (if the there are only a few result entries) or top 10% (if the there are many result entries) of the results. Where the real epitope is unknown, we would suggest running MIMOX with a range of parameters and consensus sequence derived fragments to find overlapping or otherwise promising (high surface accessibility) candidate.

### Related software comparison

As we have mentioned previously, several groups have researched algorithms and programs that may assist and automate phage display based epitope mapping. Based on the dependency on antigen structure, the existing programs can be classified into three categories. FINDMAP belongs to the first category, which is independent of any structural information. FINDMAP has been implemented as a C++ program. It aligns a probe (e.g. a consensus sequence derived from a set of mimotopes) to the sequence of native antigen, allowing any permutation of the probe sequence. It uses a two-part scoring system to evaluate the quality of alignments and a branch-and-bound algorithm to find an alignment with maximum score[13].

The programs in the second category include SiteLight, 3DEX, Mapitope, and MIMOX. SiteLight was implemented in C++ and it has been tested on Red Hat Linux. First, the program divides native protein surface into overlapping patches based on geodesic distances between residues; then aligns each mimotope in the library with each patch and scores and sorts them; finally, high scoring matches are selected iteratively until 25% of the native protein is covered[12]. Another program 3DEX was implemented in Visual Basic and could only run on Windows. It divides a sequence into a set of overlapping subsequences with a user-defined length (3-maximum length of mimotope). Then, it searches for matching residues at each position of the above subsequences against the sequence or PDB structure of native protein and links the neighbours iteratively until the first subsequence is complete. This is repeated for the following subsequences to complete the mimotope and return the result[14]. Mapitope was also implemented in C++ and its algorithm was first described by Enshell-Seijffers in 2003. Briefly, Mapitope deconvolutes a set of mimotope sequences into a set of overlapping amino acids pairs (AAP). Then a set of major statistically significant pairs (SSP) are identified based on the AAP. Later, the SSP are mapped and clustered in the antigen structure. Finally, the most elaborate and diverse clusters on the antigen surface are identified and regarded as the predicted epitope candidates[16,17].

MIMOP, a work published very recently, comprises the third category. MIMOP includes two approaches. One is called MimAlign, which can predict potential epitopic regions (PER) from mimotope and antigen sequences, and from the antigen structure if available. The other called MimCons, can predict PER from mimotope sequences but requires the antigen structure[15]. It seems that MIMOP can work with or without the antigen structure from the published case studies. However, the sequence of the only case that is independent of antigen structure is just a continuous subsequence of the antigen sequence. Thus, more studies are still needed to prove that MimAlign can work without antigen structure information.

All the existing programs described above have succeeded in given cases. However, a systematic evaluation on these tools is absent. Moreover, as shown in the Table 1, most of the existing tools have not been implemented as a publicly accessible online service until now, making it less convenient for the community to access, utilize, and evaluate them.

**Table 1:** Comparing available programs related to MIMOX

	SiteLight	FINDMAP	3DEX	MIMOP	Mapitope	MIMOX
Coded in	C++	C++	VB	PHP	C++	Perl
Operating System	Linux	Not stated	Windows	Platform independent	Windows	Platform independent
Interface	Command Line	Not stated	Graphic	Web	Command Line	Web
Online Service	no	no	no	available on request	under construction	available
Structure Dependent	yes	no	yes	yes/no	yes	yes

### Future work

Like all software, bugs will have crept into MIMOX during the programming. We expect users will send their feedback to help us maintain and improve MIMOX in the future. A new version of MIMOX with more user definable options and supporting multiple-chain antigens will be implemented in the future, allowing epitopes formed by residues from different polypeptide chains to also be predicted. A systematic evaluation and comparison study with all available tools including MIMOX that assist phage display based epitope mapping is also under our consideration.

## Conclusion

MIMOX, a web application for phage display based epitope mapping has been coded with Perl. It is helpful for molecular biologists to identify the native epitope of an antibody based on the antigen structure and a set of mimotope sequences they get through phage display technology. As a publicly accessible web tool in this area, MIMOX is very convenient for the community to access, utilize, and evaluate, complementing other existing programs.

## Availability and requirements

Project Name: MIMOX

Project Homepage: 

Operating System: Platform independent

Programming Language: Perl

Other Requirements: Java1.2 or higher

License: GNU GPL

Any Restrictions to use by non-academics: none

## Abbreviations

CGI: Common Gateway Interface; GNU: GNU's Not UNIX; GPL: General Public License; HIV: Human Immunodeficiency Virus; MIMOX: MIMOtope eXplorer; PDB: Protein Data Bank; PHP: Hypertext Preprocessor; VB: Visual Basic

## Authors' contributions

JH conceived of this study, coded the program and drafted the manuscript. AG discussed and suggested for algorithm improvement as well as helped to draft the manuscript. WH collected data and tested the program. MK supervised and directed the development process of the whole project and revised the manuscript critically. All authors have read and approved the final manuscript.

## Supplementary Material

Additional File 1 Test dataset for MIMOX. The data provided represent the cases that have been tested with MIMOX. Click here for file
